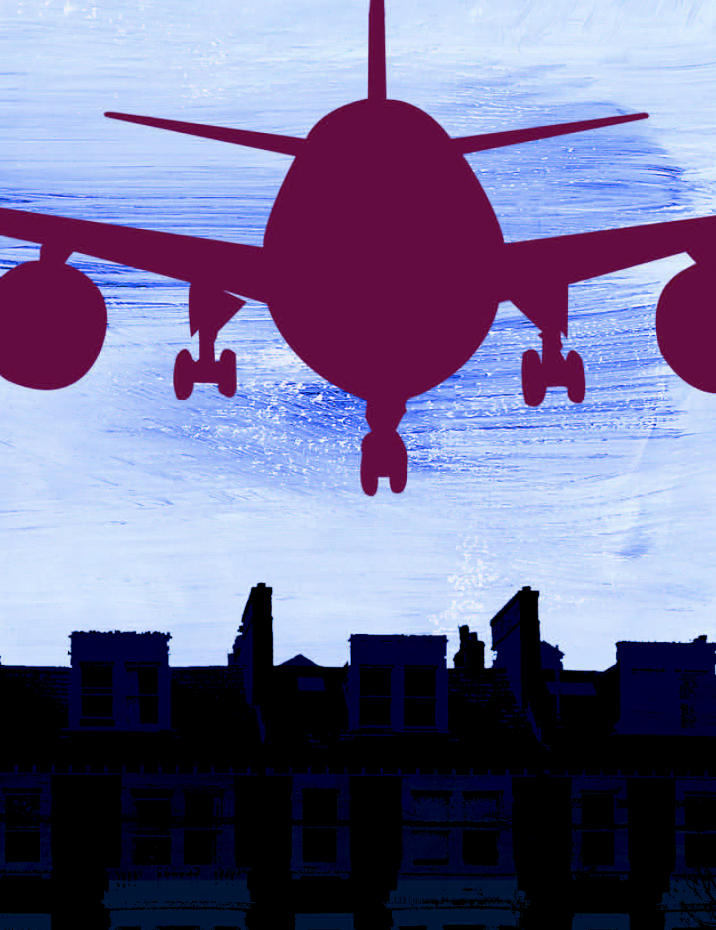# Noise that Annoys: Regulating Unwanted Sound

**DOI:** 10.1289/ehp.113-a42

**Published:** 2005-01

**Authors:** Charles W. Schmidt

Anyone who lives in Bensenville, Illinois, knows about the “Bensenville pause.” According to long-time resident Pat Johnson, it goes like this: As the roar of a jetliner departing from nearby O’Hare International Airport becomes a blasting shriek, the residents of this small town stop talking and wait. Conversations pick up as the plane goes by, but they soon pause again; planes fly over Bensenville every three to four minutes. “Sometimes, it’s hard to fall asleep,” Johnson says. “You do, but then you wake up again. The noise interrupts churches and classrooms. There are times you can’t even talk on the phone.”

The case of Bensenville may be extreme, but it’s not unusual. Today, millions of Americans suffer from noise pollution caused by planes, road traffic, car alarms, boom boxes, stereos, and many other volume-enhanced contraptions, some of them earsplitting by design. Until recently, for example, Sony Corporation marketed amplifiers and speakers with a “Disturb The Peace” advertising campaign that boasted of “new ways to offend.” Les Blomberg, who directs the nonprofit Noise Pollution Clearinghouse, refers to unwanted noise as *aural litter* or *audible trash* —“That is how people experience community noise: as someone else’s garbage thrown into their space,” he says.

In many developed countries, such as some member nations of the European Union, governments have stepped in to protect citizens from this aural assault with regulations that set maximum sound levels for construction equipment, vehicles, and airplanes. Switzerland has gone so far as to prohibit aircraft departures between 11:30 p.m. and 5:00 a.m., except in unusual and unforeseen cases. Yet Americans seeking relief from noise pollution are remarkably powerless.

## A Regulatory Void

Years ago, the Environmental Protection Agency (EPA) had federal regulatory authority over noise pollution. Working through the agency’s Office of Noise Abatement and Control (ONAC), EPA staff developed model noise codes that were provided to local municipalities upon request. With assistance from the EPA, these model codes were then customized to address local noise pollution sources and concerns. The EPA also had enforcement authority over the Noise Control Act of 1972, a national law designed to protect Americans from “noise that jeopardizes their health or welfare.”

ONAC was preparing to establish federal noise standards for transportation sources and construction machinery when its funding was abruptly cut off in 1981 by the incoming Reagan administration. With one stroke, the administration crippled the Noise Control Act and left the country without a coherent national noise policy. Reagan’s view was that noise was better managed by states and local communities. However, Blomberg says, with ONAC’s closure came cuts for federal assistance in this area. Without federal dollars, more local efforts to fight noise pollution were forced to compete forstate funding—often unsuccessfully.

Meanwhile, efforts to draft national noise standards for transportation sources—which at the time were cited by the EPA as the greatest source of residential exposure to noise pollution—were stopped in their tracks and have not been revived. Since ONAC’s closure, federal oversight of transportation noise has been filled by agencies whose core mandates are often at odds with noise control.

The Federal Aviation Administration (FAA), for instance, has the authority to determine where and how airport noise should be managed. But according to Peter Kirsch, an attorney with the Denver, Colorado–based firm Kaplan Kirsch & Rockwell who has represented plaintiffs in noise litigation, this responsibility conflicts with one of the FAA’s main purposes, which is to promote the growth of the aviation industry. Likewise, the Federal Highway Administration has primary authority over traffic noise—yet this agency’s core mission is to build, maintain, and upgrade the nation’s road system.

Consequently, communities that suffer from noise pollution are often thwarted by officials from the FAA and other agencies. Even efforts by individual airports to become more noise-friendly are usually rebuffed by FAA officials—particularly if the solutions involve flight restrictions that could impede commerce, Kirsch says. “Airports usually have to fight the feds to achieve some environmental gains,” he says. “It’s a backwards approach to environmental protection, and it creates a permanent animosity among the FAA, local communities, and airport operators.”

## A Health Problem?

Why has noise pollution—the bane of existence for so many people—been given such short shrift by the federal government? One reason is the disagreement over its inherent health risks.

Some researchers, for instance Birgitta Berglund, a professor of psychology at Stockholm University in Sweden and editor of the World Health Organization’s 1999 *Guidelines for Community Noise*, suggest unwanted sound exposure can cause hearing loss, fatigue, loss of balance, nausea, reduced sex drive, headaches, and mental disorders. Others link noise pollution with susceptibility to colds, changes in blood pressure, and heart disease.

But establishing causal links between sounds and health risks is challenging, if not impossible, says Sanford Fidell, a noise expert and a principal of Fidell Associates, a Woodland Hills, California–based consulting firm for airports, communities, and government agencies. Unlike drugs or chemicals, noise pollution leaves no residue in the body, he says. Therefore, it’s difficult to measure its cumulative effects or to distinguish noise impacts from other, similar stressors. Humans are clearly irritated by noise, but their reactions to it are tempered by personality and other idiosyncratic factors.

“One thing that’s certain is that there’s a causal link between sleep disturbance and noise,” says Eric Zwerling, director of the Rutgers University Noise Technical Assistance Center. “And there’s no question that sleep disturbance results in a loss of productivity and efficiency and a greater potential for accidents.” Zwerling says his views are backed by evidence provided by the EPA in its seminal 1974 guidance known most commonly as the “levels document.”

## The Airport Controversy

The FAA regulates noise according to a value called the day–night average sound level, abbreviated as DNL. Based on its interpretation of the scientific literature, the Federal Interagency Committee on Aviation Noise (FICAN) noted in a 1992 report titled *Federal Agency Review of Selected Airport Noise Analysis Issues* that 12.3% of residents are “highly annoyed” once noise reaches an average of 65 decibels (dB). The DNL 65 dB is now an established regulatory trigger for FAA-funded noise remediation efforts. In a standard practice, officials will designate a DNL 65 dB “contour zone” around an airport, within which residents may qualify for home buy-outs or structural soundproofing, the latter being the FAA’s preferred remedial option to mitigate noise impacts.

Many experts are critical not only of the DNL metric and the 65-dB threshold, which they view as economically motivated with little basis in science, but also of FICAN itself, which has heavy representation from the aviation industry. “You could say FICAN is the fox guarding the henhouse,” says Kirsch. He adds that the DNL 65 dB threshold is problematic because it represents flight noise averaged over a typical 24-hour period. Thus, the value doesn’t reflect much louder short-term noise events, nor does it reflect the frequency of noise events among a given population.

Caught between the regulators and the science are communities like Bensenville, which increasingly turn to the courts in search of relief. Cases like these can drag on for many years. For instance, Bensen-ville’s activists—many of them housewives and mothers—have fought O’Hare over noise, among other issues, for more than three decades.

For its part, the FAA claims to have lessened the impact of aircraft noise by requiring quieter “Stage III” engines on planes that weigh 75,000 pounds or more. The requirement for Stage III engines on larger aircraft was imposed by the Airport Noise and Capacity Act (ANCA) of 1990, which also created a mechanism for airports to follow if they wanted to restrict the remaining older, louder Stage I or II planes weighing less than 75,000 pounds. A spokesperson with the FAA Office of Public Affairs says that in 1975, with 250 million people flying a year, there were 7 million people affected by aircraft noise. Today, 700 million people fly each year, but the FAA estimates 600,000 people are affected by noise (although Blomberg says most experts outside the FAA think this number is far too low).

The validity of the FAA’s numbers has no bearing on flight frequency, which has increased 40% since 1990, according to the U.S. Bureau of Transportation Statistics. And flight frequency is among the problems most often cited by those who suffer from aircraft noise. Moreover, under ANCA, Stage III engines are not required for planes that weigh less than 75,000 pounds, which include corporate jets and other aircraft whose use is steadily rising. Kirsch is now involved in a pivotal case in Naples, Florida, where in 2001 the local airport successfully used the ANCA procedures to ban the loud Stage I and II planes that are lighter than the law’s weight limit. Ever since, Kirsch has fought a protracted legal battle with the industry and the FAA, which is struggling to overturn the ban and reintroduce the louder aircraft against the desires of both the community and the airport itself.

## A Local Choice

Transportation aside, much of the annoying racket assaulting residential eardrums comes under the purview of local ordinances. Commercial and industrial noise sources, loud music, barking dogs, early-morning lawn mowers, and unmuffled motorcycles could all be regulated if local governments so chose. The challenge is to overcome local opposition, prepare the necessary regulations, and then educate law enforcement and residential communities about their existence.

Zwerling and his staff at the Rutgers University Noise Technical Assistance Center write customized noise codes for local jurisdictions and train designated municipal officials on ways to monitor and enforce them. “It’s unbelievably gratifying,” he says.

But Zwerling concedes that transportation sources are not so easily addressed, when local regulation is prevented by federal preemption. Unlike local governments, who have no vested interest in the operation of thumping subwoofers or the First Amendment rights of a Led Zeppelin–obsessed teenager, the federal agencies that regulate transportation noise makers must by necessity be concerned with those constituents’ economic well-being.

“When it comes to noise, I think it’s important to have some distance from those who regulate and those who are regulated,” Zwerling says. “The feds need to either get all the way into regulating noise or they need to get all the way out so the locals can do it. That way, a powerful agency like the California Air Resources Board could start setting noise standards for the state. Pretty soon, other states like New York or New Jersey would follow suit. You need a state with enough power to set some influential standards.”

## Figures and Tables

**Figure:**